# Real-Time Digital Contact Tracing: Development of a System to Control COVID-19 Outbreaks in Nursing Homes and Long-Term Care Facilities

**DOI:** 10.2196/20828

**Published:** 2020-08-25

**Authors:** Gerald Wilmink, Ilyssa Summer, David Marsyla, Subhashree Sukhu, Jeffrey Grote, Gregory Zobel, Howard Fillit, Satish Movva

**Affiliations:** 1 CarePredict Plantation, FL United States; 2 Icahn School of Medicine at Mount Sinai New York, NY United States; 3 Alzheimer’s Drug Discovery Foundation New York, NY United States

**Keywords:** COVID-19, SARS-CoV-2, contact tracing, nursing homes, long term care, care homes, digital contact tracing

## Abstract

**Background:**

Severe acute respiratory syndrome coronavirus 2 (SARS-CoV-2) can spread rapidly in nursing homes and long-term care (LTC) facilities. Symptoms-based screening and manual contact tracing have limitations that render them ineffective for containing the viral spread in LTC facilities. Symptoms-based screening alone cannot identify asymptomatic people who are infected, and the viral spread is too fast in confined living quarters to be contained by slow manual contact tracing processes.

**Objective:**

We describe the development of a digital contact tracing system that LTC facilities can use to rapidly identify and contain asymptomatic and symptomatic SARS-CoV-2 infected contacts. A compartmental model was also developed to simulate disease transmission dynamics and to assess system performance versus conventional methods.

**Methods:**

We developed a compartmental model parameterized specifically to assess the coronavirus disease (COVID-19) transmission in LTC facilities. The model was used to quantify the impact of asymptomatic transmission and to assess the performance of several intervention groups to control outbreaks: no intervention, symptom mapping, polymerase chain reaction testing, and manual and digital contact tracing.

**Results:**

Our digital contact tracing system allows users to rapidly identify and then isolate close contacts, store and track infection data in a respiratory line listing tool, and identify contaminated rooms. Our simulation results indicate that the speed and efficiency of digital contact tracing contributed to superior control performance, yielding up to 52% fewer cases than conventional methods.

**Conclusions:**

Digital contact tracing systems show promise as an effective tool to control COVID-19 outbreaks in LTC facilities. As facilities prepare to relax restrictions and reopen to outside visitors, such tools will allow them to do so in a surgical, cost-effective manner that controls outbreaks while safely giving residents back the life they once had before this pandemic hit.

## Introduction

The coronavirus disease (COVID-19) is a rapidly spreading infectious disease caused by severe acute respiratory syndrome coronavirus 2 (SARS-CoV-2) [1]. A total of 4.0 million cases and 143,000 COVID-19–associated fatalities have been reported in the United States as of July 25, 2020 [2]. Residents of nursing homes and long-term care (LTC) facilities represent only 0.7% of the total US population yet account for 8% of cases and 47% of all COVID-19 fatalities in the United States [2,3]. LTC residents also exhibit an infection fatality rate of 18.6%—a rate that is 13 times higher than for the total population [2-8].

The vulnerability of LTC facilities to respiratory disease outbreaks is well documented, and several factors have contributed to the recent COVID-19 outcomes: high-risk population (the majority of LTC residents are advanced in age and have one or more underlying conditions), high-risk setting (the frequency, type, and duration of close contact between the residents and staff), and epidemiological features and transmission dynamics (people infected with SARS-CoV-2 can be infectious before showing symptoms and 40% of new COVID-19 cases are transmitted by asymptomatic cases) [9,10]. Due to these factors, symptoms-based monitoring and slow manual contact tracing methods presently used by LTC facilities have proven inadequate, and new tools are needed to better control COVID-19 outbreaks [11-13].

Advanced age and underlying comorbidities are well-established risk factors for severe COVID-19–associated illness, hospitalization, and death [14,15]. Adults 85 years and older represent 2% of the US population but have contributed to 33% of all COVID-19 deaths (Multimedia Appendix 1) [2,3,16,17]. This death rate is 613.1 (per 100,000 population), 14 times higher than the overall population rate [2,18]. The average COVID-19–associated hospitalization rate for adults 85 years and older is 607.3 (per 100,000 population), roughly 6 times higher than for the overall population [2,18]. Older adults are also disproportionally affected by chronic conditions, where 60% have two or more conditions, and such persons are known to be at an elevated risk for severe COVID-19–associated illness [19,20]. Richardson et al [21] found that 94% of patients hospitalized with COVID-19 exhibited one comorbidity, and 88% of patients exhibited two or more.

In addition to housing vulnerable residents, LTC facilities exhibit several intrinsic characteristics that make them high-risk settings conducive for the rapid spread of SARS-CoV-2 [22]. First, in LTC facilities, residents live together in close quarters, eat communal meals, and participate in many group social activities. Second, caregiving staff frequently assist residents with their activities of daily living (ADL) such as bathing, dressing, and eating. ADL assistance requires intimate resident contact, which increases the probability for transmission from an infected staff member or resident. Third, during the course of a work day, facility staff move from room-to-room to provide care for many different residents. In addition, many staff members may work at multiple facilities or home care agencies; thus, if they become infected, they can serve as potential vectors between facilities [11,12,23]. Overall, the frequency, type, and duration of contact between residents and staff has contributed to increased SARS-CoV-2 transmission both within and between facilities.

The epidemiological features, infection progression characteristics, and transmission dynamics of SARS-CoV-2 and COVID-19 have also contributed to the difficulties faced by LTC facilities to contain outbreaks. Such parameters are also fundamental to the development of accurate mathematical models, control systems, and effective infection control policies [9,14,24-27]. The SARS-CoV-2 virus is known to spread primarily person-to-person through large respiratory droplets (>5 µm) expelled when an infected symptomatic or asymptomatic person coughs, sneezes, or breathes [9,10]. Airborne virus transmission is also possible in confined, poorly ventilated environments such as LTC facilities because when an infected person speaks they can expel aerosols, tiny virus containing droplet nuclei (≤5 µm), that can linger in the air for up to 14 minutes [28-32]. SARS-CoV-2 is also believed to be viable and infectious on surfaces for hours; therefore, transmission may occur indirectly via *fomites*, contamination of surfaces in the environment [33,34].

**Figure 1:**
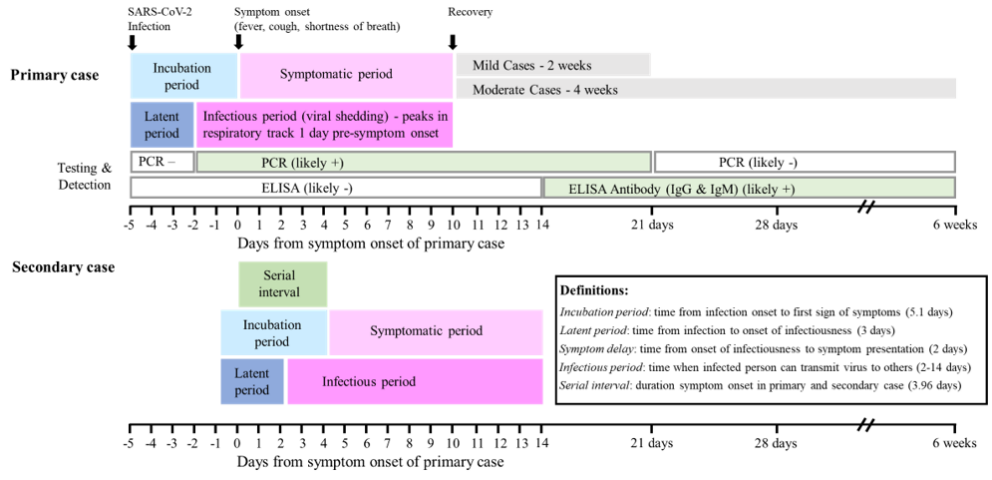
Overview of current estimates on key epidemiological features, infection characteristics, transmission dynamics, and testing methods for SARS-CoV-2 and the coronavirus disease. ELISA: enzyme-linked immunosorbent assay; IgG: immunoglobulin G; IgM: immunoglobulin M; PCR: polymerase chain reaction; SARS-CoV-2: severe acute respiratory syndrome coronavirus 2.

Isolation of confirmed and suspected cases, and identification of contacts via contact tracing are crucial to effective control efforts. These methods hinge on three key epidemiological parameters: (1) basic reproduction number (R_0_), the average number of secondary infections generated by each infection; (2) serial interval, duration between successive infections and speed of viral spread; and (3) proportion of asymptomatic transmission. Best estimates indicate that the R_0_ for SARS-CoV-2 causing COVID-19 is 2.5, which is significantly higher than the flu [35]. The serial interval, duration between symptom onset in a primary and secondary case, is estimated to be 3.96 days, which is almost twice as fast as SARS-CoV-1 [26,27]. The mean latent period, time from infection to onset of infectiousness, is estimated to be 3 days, which is shorter than the 5.1 day incubation period, time between infection and onset of symptoms (fever, cough, shortness of breath; Figure 1) [9,14,24-27]. Consequently, people infected with SARS-CoV-2 are most infectious 1-3 days before showing symptoms and up to 10 days after symptom onset [14,25]. SARS-CoV-2 is transmitted via symptomatic, asymptomatic, and presymptomatic routes, and current best estimates indicate the following: 25%-81% of cases are asymptomatic [36-38], symptomatic and asymptomatic cases are equally infectious [35], and 40%-44% of new COVID-19 cases are transmitted from presymptomatic individuals [14,35,36,39,40]. These features are consistent with early reports from LTC facilities, where 56%-73% of residents that tested positive for COVID-19 were asymptomatic at the time of testing [11,12,41] and that both presymptomatic and asymptomatic cases contributed to rapid facility spread [11-13]. Thus, symptom-based screening alone failed to detect asymptomatic infectious cases, and Arons et al [11] posited that conventional screening approaches in LTC facilities are inadequate because symptoms-based screening and polymerase chain reaction (PCR) tests are only being performed on symptomatic persons [12,13]. LTC facilities need contact tracing systems to rapidly identify, contain, and then broadly test asymptomatic infectious contacts [42].

Contact tracing, a core disease control measure used by public health authorities (PHAs) to prevent the spread of infectious diseases, is now being employed to identify and isolate individuals that came in close contact with a person infected with SARS-CoV-2 [43]. The manual contact tracing process is slow and has inherent time delays between confirming a case and finding a person’s contacts [9,44,45]. These time delays give secondary contacts more time to transmit the virus even further in the facility. Manual contact tracing also relies on humans both for data collection and data entry, which increases the potential for inaccurate or incomplete results due to human error. For the tracing process, a case needs to remember and report all contacts made over the past 14 days. In the LTC setting, an infected resident may have 10-30 close contacts, and older adults that may be experiencing memory impairment or dementia may forget their close contacts. Since more than 70% of contacts must be traced to control an outbreak [46], this may be difficult to achieve using manual contact tracing in a LTC facility.

Since SARS-CoV-2 spreads too fast to be contained by slow manual contact tracing, several digital contact tracing tools using smartphone-based apps have been developed [47,48]. If widely adopted, these apps show promise to effectively mitigate the spread of SARS-CoV-2 for the general population; however, smartphone-based contact tracing may have limited utility in LTC facilities for several reasons. First, LTC residents are typically older adults, and only 17% of adults 80 years and older own a smartphone [49]. Second, staff in many LTC facilities are not permitted to use a smartphone during the work day. Finally, smartphone-based approaches use Bluetooth technology, which transmits through thin walls in a facility and can result in false positives. Due to these limitations, there is benefit to having a digital contact tracing system built specifically for use in LTC facilities.

In this study we describe the development and implementation of a real-time digital contact tracing system designed specifically for LTC facilities to mitigate the spread of SARS-CoV-2 infections. Additionally, we developed a new susceptible-exposed-infectious-recovered (SEIR)–type infectious model that was adopted and parameterized specifically to describe propagation of COVID-19 in LTC facilities. The model was also used to simulate and assess the interventional performance of digital contact tracing compared to symptom-based mapping, manual contact tracing, and PCR testing.

## Methods

### Real-Time Digital Contact Tracing System

**Figure 2:**
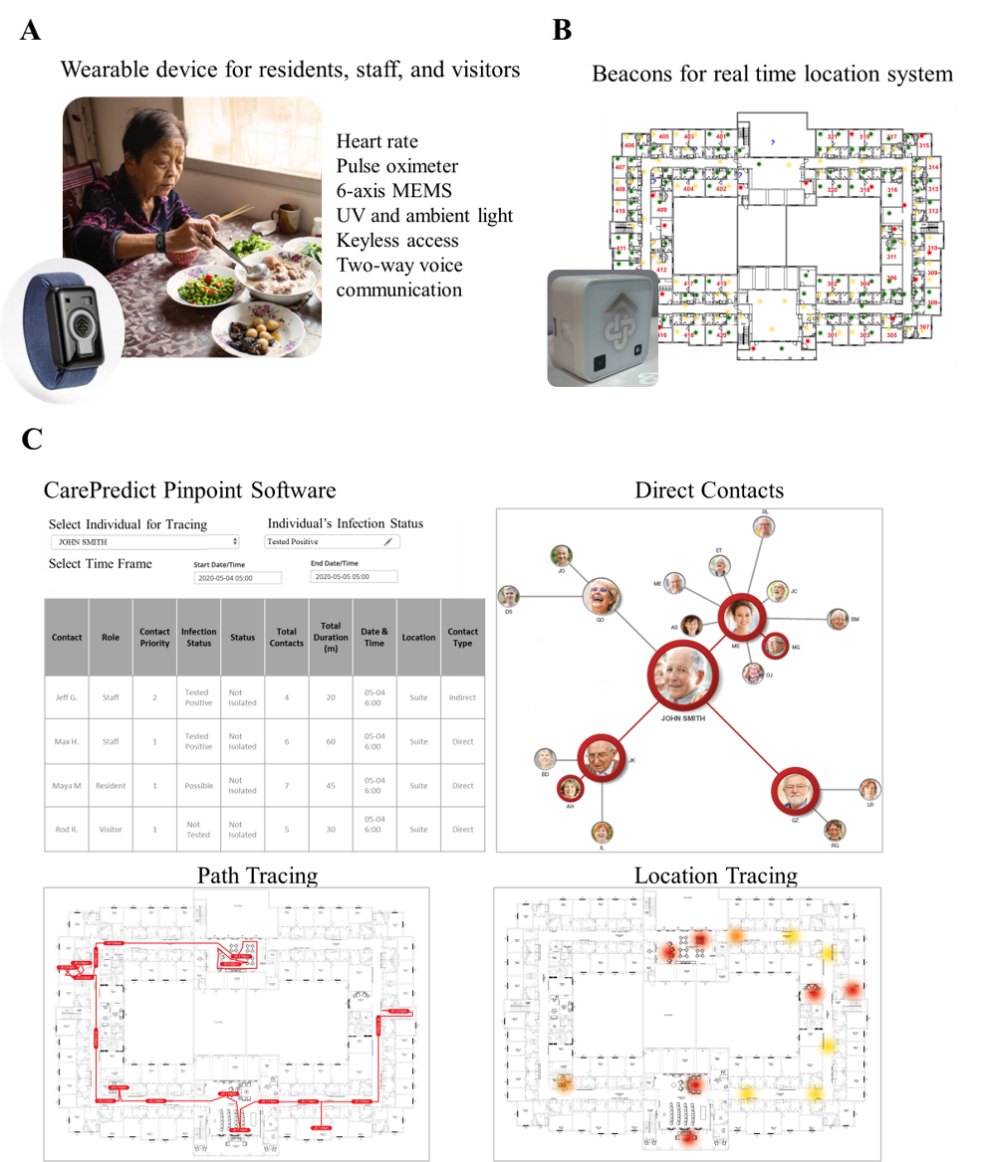
Digital contact tracing system: wearable device, real-time location tracking, and software. A: wearable device; B: real-time location system for retrospective contact tracing; C: PinPoint software. MEMS: microelectromechanical systems.

The CarePredict PinPoint is a real-time digital contact tracing system designed for use in an LTC facility. The system is used to rapidly identify and categorize individuals (staff, residents, and visitors) that may have been exposed to a person infected with COVID-19. The system consists of a wrist-worn wearable device (Tempo), beacons for real-time location tracking, and a cloud-based software application for visualization of egocentric contact networks (Figure 2) [50].

The wearable is worn on the dominant arm of residents, staff, and visitors. The wearable recognizes gestures according to the changes in the user’s wrist kinematics and autonomously provides outputs on the user’s ADL such as eating, bathing, walking, bathroom visits, and sleep duration. The wearable houses the following sensors for detection of the user’s heart rate, blood oxygenation (via pulse oximetry), 6-axis microelectromechanical systems sensor, and UV and ambient light sensors (Figure 2A). When coupled with data from context beacons, indoor positioning information is obtained such as the type of room in which the person is located (Figure 2B). The wearable uses Wi-Fi to communicate data to the cloud over an encrypted connection and supports two-way audio so the wearer can communicate via mobile apps on iOS and Android devices. The device supports radio-frequency identification (RFID) protocols for integration with electronic door access systems. The wearable measures 50 x 33 x 17.7 mm; weighs 40 grams; and includes a microprocessor, RFID, Bluetooth 4, and Wi-Fi 802.11 b/g/n. The wearable uses a 380mAH Li-ion 10.6g polymer battery, which has 50-100 hours of battery life. The device uses a swappable battery design so the user does not have to take off the device for charging. The wearable has an operational temperature range of –20 °C to 55 °C, water-resistant to IP67, and has the following certifications: FCC (Federal Communications Commission), CE (Conformité Européenne), TELEC (Telecom Engineering Center), and Bluetooth.

The real-time location system uses beacons to determine the room-level indoor location of the wearable, and the duration of contact with other wearable devices. The beacon measures 52.1 x 52.1 x 28.0 mm, weighs 78 g, and uses Lithium CR123A batteries. A patented line-of-sight technology is used for multi-floor level indoor positioning with room-level accuracy and no bleed-throughs.

The PinPoint software consists of three tools (Figure 2C):

1. Contact tracing workspace: direct—identify all individuals the infected person (person under investigation [PUI]) had direct contact with in the facility; secondary or indirect contacts (individuals who subsequently came in contact with the PUIs direct contacts); and environmental (individuals who spent time in facility rooms that may have been contaminated by the PUI [ie, possible fomite or aerosol transmission]). Each unique interaction is summarized regarding the time of day, duration, and location. All three types of contacts are then classified as priority 1 or priority 2 contacts (Figure 2C).
2. Line listing tool: digitized respiratory line listing tool to store and track infection data
3. Decontamination tool: identify all of the confined areas (suites, bathrooms, offices) and common areas that the PUI visited in the facility—including the day, time, and duration. The high-touch surfaces in these rooms can then be cleaned and disinfected.

### Simulation Model

We developed a specialized *SEIR*-type compartmental model to simulate the dynamics of propagation, disease transmission, and containment of SARS-CoV-2 cases in LTC facilities [51,52]. In this model, individuals within the LTC facility (residents and staff) are separated into mutually exclusive groups, or compartments, based on their disease state: susceptible (*S*), exposed (*E*), infected (*I*), quarantined (*Q*), recovered (*R*), and deceased (*D*). Infected individuals were further segmented into two distinct groups: presymptomatic (*I_P_*) and symptomatic infectious individuals (*I_S_*). The decoupled compartments include deceased (*D*) and quarantined individuals (*Q*) from the (*E, I_P_, or I_S_*) compartments. The model assumes no demography, such that the population size is constant, denoted by *N*. The facility was assumed to have a population of 120 persons, consisting of 80 residents and 40 staff. A schematic representation of the model is provided in Multimedia Appendix 2 [51]. The population dynamics are modeled by the following system of differential equations:

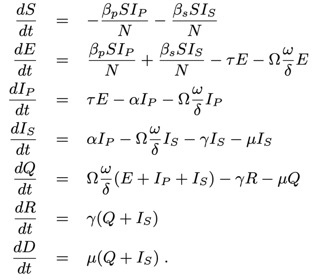



where *N* = *S* + *E* + *I_P_* + *I_S_* + *Q* + *R* + *D*.

The transmission parameters, *β_p_* and *β_s_*, represent the transmission rate for presymptomatic and symptomatic individuals; *τ* is the mean latent period; *α* is the difference in latent and incubation period, where *α* = (incubation period – *τ*). The following parameters varied depending on the intervention approach: Ω*_i_* is an intervention on/off parameter; *ω* is the intervention traced contact probability; *δ* is the time delay to trace, where *ω/δ* is the rate at which a contact trace is quarantined; and *µ* is the death rate. For this model, we assumed that once an individual is quarantined, all staff wear personal protective equipment when interacting with residents, and thus, no further transmission would occur between quarantined and susceptible individuals.

The model was developed to assess the performance, defined as the number of cases and resultant deaths, for several intervention types: digital contact tracing, manual contact tracing, symptom-based mapping, PCR testing, and no intervention. Table 1 contains the intervention parameters and assumptions used in the model. For no intervention, *β* is set to average contacts per day from the facility. For intervention, *β_s_* = *β_p_* */* 2. For symptom mapping, we assume that only symptomatic individuals are quarantined but presymptomatic individuals are not (Ω=0). The initial time delays (*δ)* for each intervention method were as follows: symptom-based mapping (1 day), manual contact tracing (2 days), swab PCR (1 day), and digital contact tracing (0.1 days). Simulations were also conducted where the time delay parameter was adjusted to assess the impact that time delay has on interventional performance.

**Table 1:** Parameters for compartmental infection and intervention model.

Name and symbol		Description	Central value	Range	References
Transmission rate (presymptomatic) (*β*_p_)		Infectious transmission rate for presymptomatic individuals	0.52 day^−1^	0.5-1.5 day^−1^	[53], fit data [13], [35]
Transmission rate (symptomatic) (*β*_s_)		Infectious transmission rate for symptomatic individuals. Assume half the contacts.	*β*_p_*/*2 day^−1^	0.5-1.5 day^−1^	[13,53], [35]
Latency period (*τ*)		Time from infection to infectious	4 days	3-5 days	[9,14,24-27]
Incubation period (*α*)		Time from infection to symptomatic	8 days	2-14 days	[9,14,24-27]
Death rate (*µ*)		Death rate	0.02 days	0.001-0.1	[35]
**Intervention function target (Ω_i_)**					
	Manual contact tracing		1	N/A^a^	N/A
	Swab PCR^b^ testing		1: *I*_P_ *& I*_S_, 0: *E*	N/A	N/A
	Digital contact tracing		1	N/A	N/A
	Symptom mapping		0	N/A	N/A
	No intervention		0	N/A	N/A
Symptom mapping trace rate (*ω*_s_)		Probability of traced contact by tracing symptomatic individuals	0.6	N/A	[35]
Manual contact tracing rate (*Ω*_m_)		Probability of traced contact by tracing symptomatic individuals	0.7	N/A	[54]
Swab PCR testing rate (*Ω*_m_)		Probability of traced contact by tracing symptomatic individuals	0.7	N/A	[35]
Digital contact tracing rate (*ω*_d_)		Probability of traced contact individuals	0.9	N/A	This study
**Time delay to trace (*δ*)**					
	Symptom-based mapping		1 day	1-4 days	[11-13]
	Manual contact tracing		2 days	1-4 days	[44]
	PCR test		1 days	1-6 days	[24]
	Digital contact tracing		2.4 hours	N/A	This study

^a^N/A: not applicable.

^b^PCR: polymerase chain reaction,

## Results

### System Implementation

**Figure 3:**
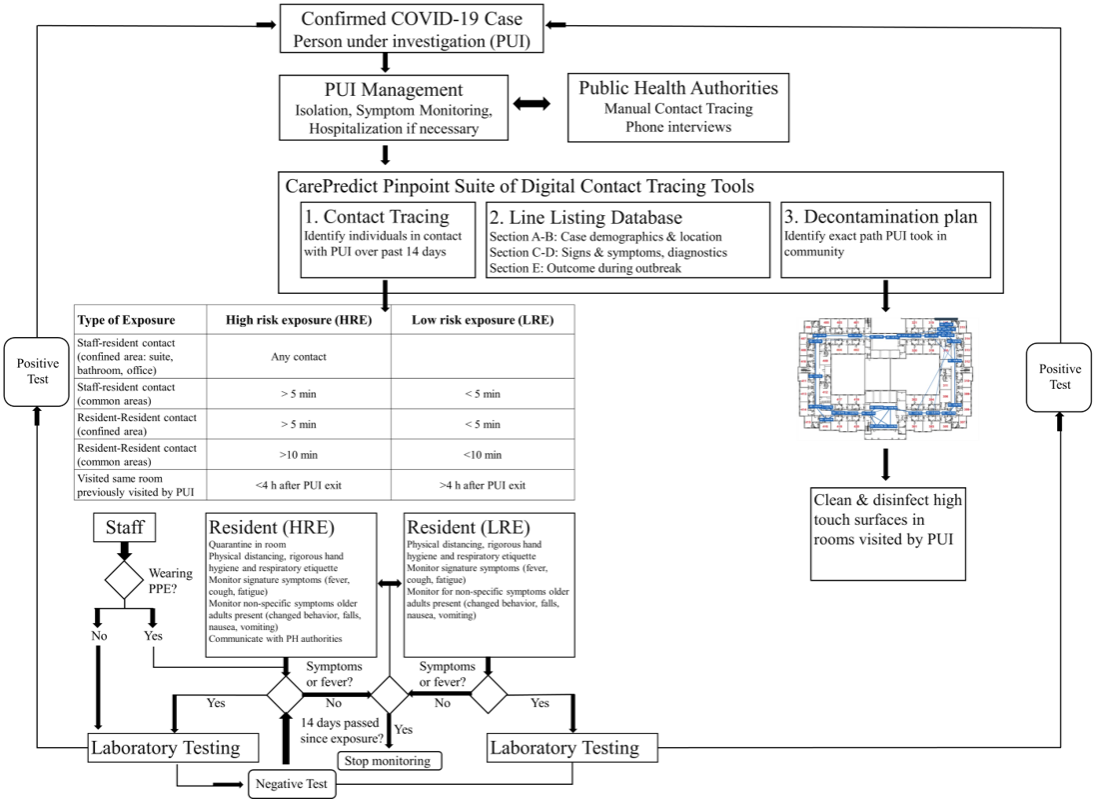
Sample representation for integrating CarePredict’s PinPoint system and software into a long-term care facility's COVID-19 risk assessment workflow. General workflow diagram developed to be consistent with those proposed by the European Centre for Disease Prevention and Control. COVID-19: coronavirus disease; PH: public health; PPE: personal protective equipment; PUI: person under investigation.

An example of implementation and workflow for the CarePredict PinPoint digital contact tracing system is provided in Figure 3 [44]. The process could work in the following manner. First, a positive COVID-19 case, defined as a PUI is confirmed, immediately isolated, has symptoms monitored, and is hospitalized if necessary. Data for the PUI would then be inputted into the Pinpoint software respiratory line listing tool: A. case demographic; B. case location; C. signs and symptoms; D. diagnostics; and E. outcome during outbreak. This line list date is then provided to the PHAs so they can begin manual contact tracing processes. The digital contact tracing tool would then be executed to identify the individuals that came in contact with the PUI over the past 14 days. The contacts are classified as either priority 1 (high-risk exposures) or priority 2 (low-risk exposures), and staff would provide the necessary next steps of care. The priority 1 contacts would be immediately quarantined and their symptoms monitored, and the priority 2 contacts would be monitored and provided safety instructions regarding physical distancing, rigorous hand hygiene, and respiratory etiquette. For safety precautions, the temperature of all contacts would be measured to see if the person had a fever [55]. If signature or nonspecific symptoms are not observed for 14 days then monitoring is stopped. PCR testing should be conducted on all exposed contacts (both symptomatic and asymptomatic) to determine if infected by SARS-CoV-2 or another pathogen. After completing the contact tracing runs, the decontamination tool would be used to determine the rooms and areas in the facility that may be in infected and require cleaning.

### Simulation Model

**Figure 4:**
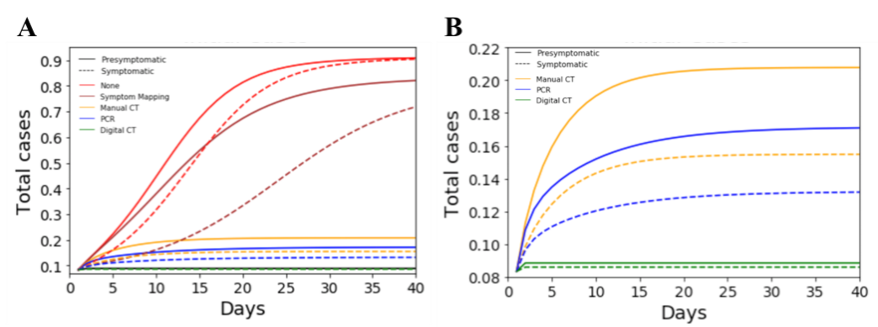
Assessing the impact of presymptomatic cases on facility spread. Simulations were performed to compare transmission and interventional control for two initial seeding conditions: presymptomatic (filled colored lines: 10 presymptomatic and 0 symptomatic cases) and symptomatic (dotted colored lines: 0 presymptomatic and 10 symptomatic cases). Simulations were performed to measure the number of total cases as a function of time for each intervention group: digital contact tracing, PCR testing, manual contact tracing, symptom-based monitoring, and no intervention. A: total cases over time for each intervention group and initial seeding condition. B. Total cases over time for manual contact tracing, PCR testing, and digital contact tracing. CT: contact tracing; PCR: polymerase chain reaction.

Asymptomatic SARS-CoV-2 infected cases contributed to the rapid spread in several LTC facilities, and conventional methods were inadequate to control those outbreaks [11,12]. To assess the impact that presymptomatic cases have on facility spread, we used our model to simulate and compare community transmission for two initial conditions: one seeded with 10 presymptomatic cases and the other seeded with 10 symptomatic cases. Simulation results for each intervention group are presented in Figure 4A. For all intervention groups, the seeding of presymptomatic cases (full lines) resulted in 6%-10% more total cases (ie, greater infection spread) than the group seeded with symptomatic cases (dotted lines). Symptom-based monitoring alone was the least effective control method, yielding 60%-71% more cases than the other interventional groups. Digital contact tracing provided the most effective intervention control. Five days after presymptomatic seeding, digital contact tracing yielded 5% and 7% fewer cases than PCR testing and manual contact tracing, respectively. After 40 days, the digital contact tracing provided 6% and 12% fewer cases than PCR testing and manual contact tracing, respectively (Figure 4B).

**Figure 5:**
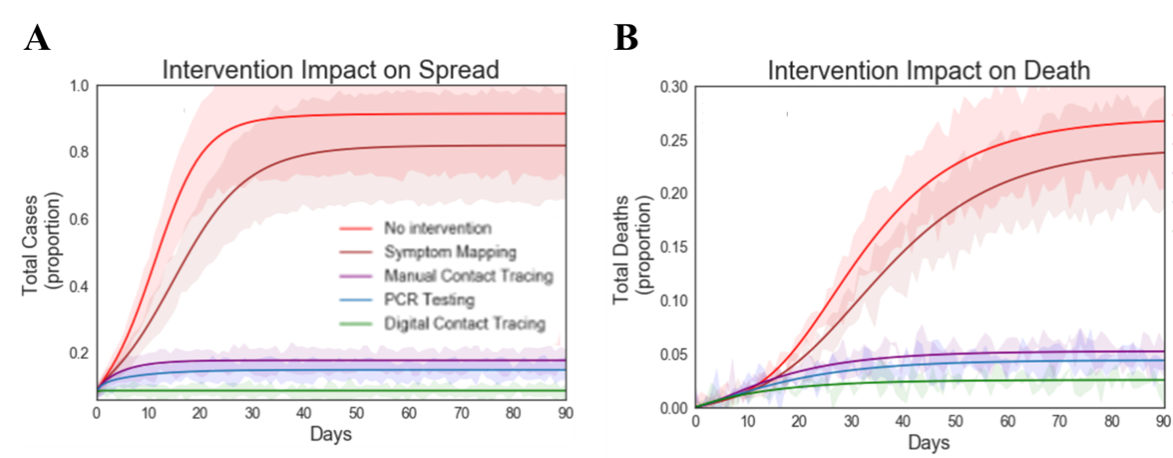
Quantifying control success for each intervention group. A: total cases (proportion) over time. B: total deaths (proportion) over time. Simulations were performed for all intervention groups using initial seeding conditions: 10 cases (40% presymptomatic and 60% symptomatic cases). Time delay to trace for digital contact tracing (0.1 days), symptom-based mapping (1 day), manual contact tracing (2 days), and PCR testing (1 day). Simulations were performed to measure the total cases and deaths as a function of time for each intervention group: digital contact tracing, PCR testing, manual contact tracing, symptom-based monitoring, and no intervention. PCR: polymerase chain reaction.

To quantify control success for each intervention group, simulations were performed using an initial seeding condition of 10 cases, 40% asymptomatic and 60% symptomatic cases [35]. These conditions were selected based on current best estimates provided by the Centers for Disease Control and Prevention [35]. The simulation results for each intervention group are presented in Figure 5. Symptom-based monitoring alone was the least effective intervention method, resulting in nearly 60% more cases than the other interventional groups (Figure 5A). Digital contact tracing provided the most effective intervention control, resulting in the fewest number of new cases and deaths (Figure 5B). Direct contact tracing achieved 22%, 3%, and 2% fewer deaths than symptom-based monitoring, manual contact tracing, and PCR testing methods, respectively. The data shows that with no intervention, 26% of the total cases result in death, which is consistent with observed case infection fatalities in LTC facilities [3].

**Figure 6:**
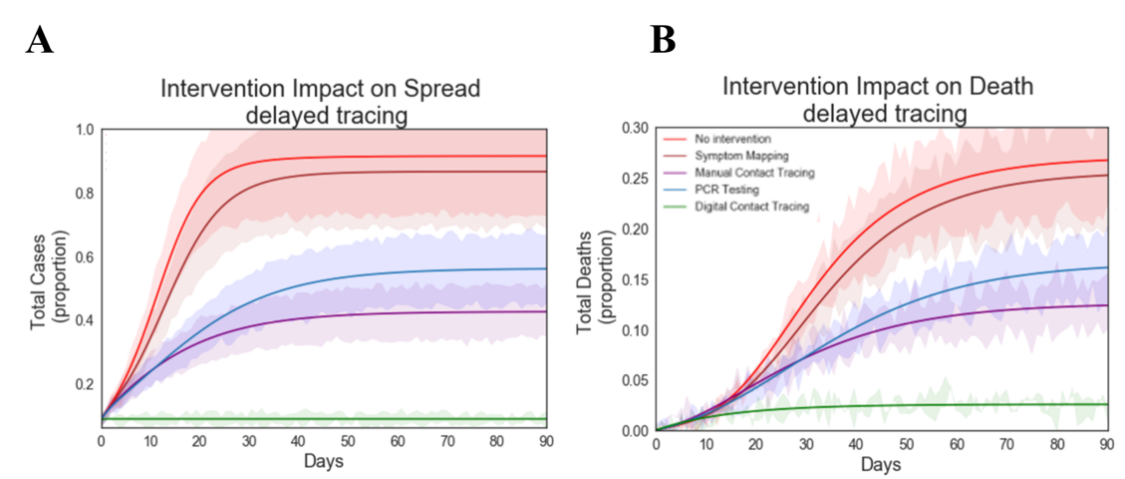
Effect of tracing delays on intervention performance. A: total cases (proportion) over time. B: total deaths (proportion) over time. Simulations were performed for all intervention groups using initial seeding conditions: 10 cases (40% presymptomatic and 60% symptomatic cases). Time delay to trace for digital contact tracing (0.1 days), symptom-based mapping (3 days), manual contact tracing (4 days), and PCR testing (3 days). Simulations were performed to measure the number of total cases and deaths as a function of time for all intervention groups: digital contact tracing, PCR testing, manual contact tracing, symptom-based monitoring, and no intervention. PCR: polymerase chain reaction.

Digital contact tracing software has negligible time delays as it requires minimal human resources to instantaneously execute. However, symptom-based mapping, manual contact tracing, and PCR testing are labor intensive and have intrinsic time delays. In previous simulations, we optimistically assumed that symptom-based mapping, manual contact tracing, and PCR testing could be performed quickly with time delays of 1 day, 2 days, and 1 day, respectively. To assess the impact that delayed tracing has on intervention success, we conducted simulations where we delayed the tracing time for each group by 2 days (Figure 6). The data shows that the increased delays in time to trace resulted in increases in cases and deaths for all intervention groups. Due to the increased delays, PCR testing is now less effective than manual contact tracing. This result underscores the importance of speed and rapid turnaround times. Exposed individuals’ PCR tests typically are not positive during their latency period; thus, multiple follow-up tests must be performed to ensure they are positive COVID-19 cases. Thus, if only individuals with positive PCR test results are being isolated, then the cases that are infected, not yet infectious, and not quarantined could continue to infect others in the facility.

**Figure 7:**
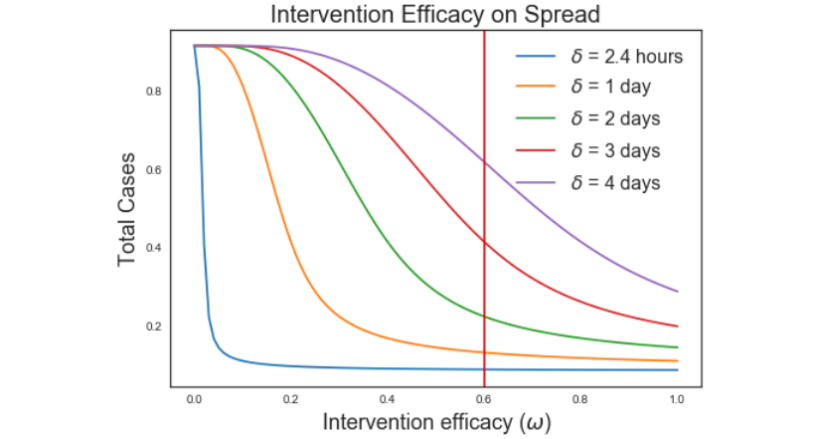
Impact of intervention efficacy and delay time on intervention success.

A series of simulations were performed to understand the impact that intervention efficacy (probability of tracing a contact) and delay have on control success (Figure 7). The data shows that as the intervention efficacy (*Ω*) increases from 0 to 0.6, the number of cases drops sharply from 1.0 to 0.15. The data shows that once an efficacy of 60% is achieved, only modest improvements in control can be achieved by improving the intervention efficacy. To assess the impact that intervention delay has on spread, simulations were conducted varying the time delay from 2.4 hours to 4 days and assuming all interventions had an intervention efficacy of 60%. The data shows that increases in delay intervention time result in sharp increases in the number of total cases. Increasing the delay time from 2.4 hours to 1 day, 2 days, 3 days, and 4 days resulted in increases in total cases by 4%, 13%, 32%, and 52%, respectively. Clearly the delay time has significantly more impact on performance than interventional efficacy.

## Discussion

Between March and July 2020, over 13,000 LTC facilities in the United States reported COVID-19 cases [56]. Many of these LTC facilities have experienced uncontrollable outbreaks resulting from the rapid and widespread transmission of SARS-CoV-2 [11,12,23,57]. As a result, residents of LTC facilities have been disproportionally impacted by SARS-CoV-2 and have accounted for over 40% of all COVID-19 fatalities worldwide [3,16]. Symptoms-based monitoring including temperature assessment [58] fails to identify asymptomatic infectious cases, and slow manual contact tracing methods have proven inadequate for controlling SARS-CoV-2 transmission in LTC facilities [11-13,23,42,59]. In this study, we describe the development of a new digital contact tracing system designed for use in LTC facilities. Our computer simulation results comparing different intervention approaches suggests that this system shows promise to be an effective tool to control COVID-19 outbreaks in LTC facilities.

In this study, we developed an epidemic compartmental model that was specifically parameterized to quantify SARS-CoV-2 transmission and control in LTC facilities. We used the model and considered various scenarios to assess the effectiveness of several intervention groups to control outbreaks: no intervention, symptom-based monitoring, PCR testing, manual contact tracing, and digital contact tracing. Under all conditions tested, the digital contact tracing system outperformed all intervention groups, achieving reduced SARS-CoV-2 spread, fewer total cases, and fewer fatalities. Most importantly, we show that the time delay is the most critical and sensitive parameter of the model. All conventional control methods (symptom-based monitoring, manual contact tracing, and PCR testing) except digital contact tracing have intrinsic time delays that cannot be compensated for with increases in efficiency. We conducted several simulations where we increased each interventional group’s probability of tracing a contact, and the results indicated that the control performance still could not reach the level achieved by digital contact tracing. Thus, the primary advantage of automated digital contact tracing methods is the speed at which potentially infectious contacts (both symptomatic and asymptomatic) can be instantly identified, classified, isolated, and tested. Given the high proportion of asymptomatic infections, the ability to quickly identify and test potentially infected persons before they show symptoms is key to preventing future transmission in LTC facilities.

Results from our simulations indicate that symptom-based screening alone was the least effective intervention group, resulting in 60%-71% greater cases and 10%-20% more deaths than the other methods. A limitation of symptoms-based monitoring methods such as temperature monitoring for a fever is that subclinical or presymptomatic secondary cases are missed. In LTC facilities, asymptomatic cases are equally prevalent and infectious as symptomatic cases and, thus, can be major contributors to COVID-19 outbreaks in LTC facilities [11,12,14,25,41]. Our data also suggests that symptom-based monitoring alone has intrinsic time delays due to the time required for people who are infected to both exhibit symptoms and then be identified by facility staff. To complicate matters, evidence is emerging that many older adults may not actually present the signature COVID-19 symptoms (ie, fever, cough, shortness of breath) [12,15,60]. Due to their blunted immune response systems or underlying chronic conditions, which may mask fever and acute illness, older adults may present atypical, nonspecific symptoms when ill with COVID-19, including increased falls, changes in activity and behavior (such as sleeping more and eating less), impaired mobility, malaise, fatigue, nausea, and even vomiting [12,15]. Thus, staff may require more time and use lower thresholds for suspicion to identify infected older adults that exhibit subtle symptoms. Such delays may translate into further spread of infection in the facility.

Manual contact tracing is a useful core disease control that is a key part of our country’s multipronged approach to mitigate COVID-19 transmission [43]. Estimates indicate that a large workforce of 300,000 tracers will be required for adequate tracing in the United States (nearly 1 tracer per 1000 people) [61]. The manual tracing process is error prone and slow because it requires a human tracer to interview new cases (~2 hours/interview) and then list, classify, and follow up with each contact (~1 hour/call/contact). Results from our simulations indicate that the time delays created by manual processes render the method less effective in LTC facilities than digital contact tracing methods. We found that digital contact tracing methods resulted in 12% fewer cases and 3% fewer deaths than manual contact tracing. As a result, manual contact tracing approaches will need to be supplemented with other rapid and efficient control measures. There are several additional challenges with using manual contact tracing alone in the LTC setting. First, an infected resident or staff member may have 10-30 close contacts, and estimates indicate that between 6 and 15 tracers require 12-24 hours to fully trace one case [44,45,62]. The delays created by this process give secondary contacts more time to transmit the virus even further in the facility. Second, manual contact tracing relies on humans both for data collection and data entry. This increases the potential for inaccurate or incomplete results due to human error. Accurate manual contact tracing requires the case to remember and report all contacts made over the past 14 days. In the LTC setting, many of the residents may have memory impairment or dementia, and thus, they may forget their contacts. The digital contact tracing system described in this study can automatically identify all of the contacts for a case and can be used to help augment manual contact tracing efforts performed by PHAs.

The most commonly used and reliable test for diagnosing SARS-CoV-2 infected cases is the reverse transcription-PCR (RT-PCR) test. PCR tests measure viral RNA and are performed using a nasopharyngeal, throat or saliva swabs, and take 1-2 days to process. PCR tests can effectively measure infection in people who are symptomatic with COVID-19 but are less likely to detect infection during the case’s latent period when they are presymptomatic [14,24]. The results from our simulation indicate that PCR testing can be an effective control method for rapidly identifying infection and minimizing transmission. However, for PCR testing to be effective, testing needs to be implemented on both symptomatic and asymptomatic exposed contacts on a universal and serial (weekly or daily) basis. In a recent study, Dora et al [41] investigated the benefit of serial RT-PCR testing of residents and staff at an LTC facility after an initial COVID-19 case was diagnosed. In this study, they found that after the first positive case was identified, 19 residents tested positive for SARS-CoV-2 and 73% were asymptomatic. All of the positive cases were rapidly transferred to an isolated ward to successfully break the chain of transmission [41]. One issue with daily universal testing at a LTC facility is the expense. PCR tests are expensive (US $150 per test), so daily testing at a 120 bed facility would cost US $18,000. Frequent PCR testing for all nursing home and LTC residents is reported to be unsustainable, where one-time tests would cost the industry US $672 million [63]. To address this challenge, many LTC facilities to date have performed PCR tests only on symptomatic COVID-19 cases. Given the high proportion of asymptomatic cases, we propose that digital contact tracing systems could be used to identify all high priority possibly infectious contacts that should be selected for PCR testing. This approach would be a cost-effective and effective method to control COVID-19 outbreaks.

### Limitations

There are several limitations of this study. First, the computational models that we developed did not incorporate the potential contribution that an individual’s underlying health conditions may have on SARS-COV-2 infection, transmission parameters, and death rate. Since the impact of such conditions is not well characterized, and empirical data is currently not available, we were unable to include these impacts in the model. However, it is well established that older adults are disproportionally affected by chronic conditions, and when such persons are infected, they have more severe COVID-19–associated illness [19,20]. Richardson et al [21] found that 94% of patients hospitalized with COVID-19 exhibited one comorbidity, and studies indicate that 94% of COVID-19 patient deaths, 78% of intensive care unit (ICU) admissions, and 71% of non-ICU hospitalizations had at least one comorbidity [64]. The most common comorbidities contributing to hospitalization were hypertension (56.6%), obesity (41.7%), and diabetes (33.8%) [21]. Studies on the effect of multiple comorbidities on adults 85 years and older indicated the following: for COVID-19 hospitalizations, comorbidities included hypertension (38%), diabetes and hypertension (22%), and chronic obstructive pulmonary disease (COPD) and hypertension (10%), and for COVID-19 deaths, comorbities included hypertension (37%); diabetes and hypertension (23%); COPD and hypertension (9%); and COPD, diabetes, and hypertension (8%) [65]. It is entirely possible that older adults with specific underlying comorbidities or a combination of particular comorbidities may exhibit varying infection, transmission, and death rates. As more data becomes available and these relationships are better characterized, we plan to incorporate these relationships into the models that we develop and test in future studies.

Second, the digital contact tracing system described in this paper is currently in use by several LTC facilities in the United States. These facilities are reporting early control success with the system [66]; however, a large enough sample size of empirical data has not been collected to date. Thus, the preliminary empirical results were not compared to those generated with our computer simulation models. Once a sufficient sample size of empirical data is collected using this system at various LTC facilities, we plan to conduct future studies to compare these findings versus the results generated by computer simulation models.

### Conclusion

Our digital contact tracing system allows users to rapidly identify and then isolate close contacts, to store and track infection data in a respiratory line listing tool, and to identify contaminated rooms. Our simulation results suggest that digital contact tracing allows for rapid and effective identification and containment of potentially infected close contacts. This digital contact tracing system shows promise as an effective tool to control COVID-19 outbreaks. At the beginning of this pandemic, many facilities implemented strict lockdown measures, which included prohibiting outside family visitors, closing community dining rooms, and reducing social activities and events. These measures were required at the time, but they negatively impacted many resident’s physical, social, psychological, and emotional health. As facilities prepare to reopen to outside visitors in the upcoming months, digital contact tracing systems will allow them to do so in a surgical, cost-effective manner that both controls outbreaks while safely giving residents back the life they once had before this pandemic hit.

## Appendix

Multimedia Appendix 1 Coronavirus disease (COVID-19) deaths. A: percentage of COVID-19 deaths by age group. B: percentage of COVID-19 deaths per state in long-term care. C: percentage of COVID-19 deaths per country in long-term care. Data as of July 24, 2020.

Multimedia Appendix 2 Schematic representation of the infection and intervention model for the coronavirus disease in long-term care facilities.

## Abbreviations

ADL: activities of daily living
CE: Conformité Européenne
COPD: chronic obstructive pulmonary disease
COVID-19: coronavirus disease
D: deceased
E: exposed
FCC: Federal Communications Commission
I: infected
ICU: intensive care unit
I_P_: presymptomatic
I_S_: symptomatic infectious individuals
LTC: long-term care
MEMS: microelectromechanical systems
PCR: polymerase chain reaction
PHA: public health authorities
PUI: person under investigation
Q: quarantined
R: recovered
RFID: radio-frequency identification
RT-PCR: reverse transcription-polymerase chain reaction
R_0_: basic reproduction number
S: susceptible
SARS-CoV-2: severe acute respiratory syndrome coronavirus 2
SEIR: susceptible-exposed-infectious-recovered
TELEC: Telecom Engineering Center
