# Supplementary figures and images for: Real-Time Digital Contact Tracing: Development of a System to Control COVID-19 Outbreaks in Nursing Homes and Long-Term Care Facilities
Source: JMIR Public Health Surveill. 2020 Aug 25;6(3):e20828. doi: 10.2196/20828 (PMC7451111; doi:10.2196/20828)

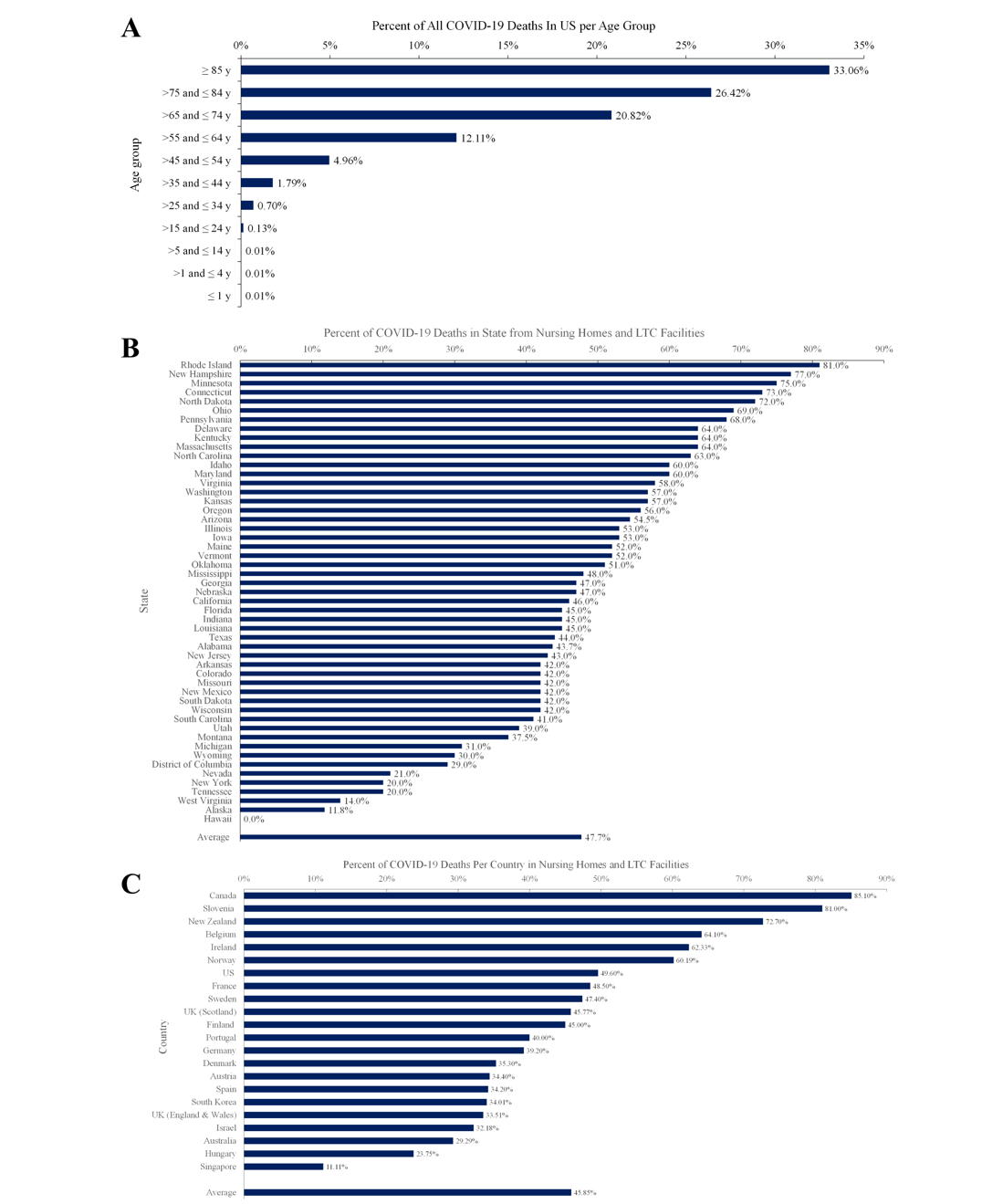

Supplement: Multimedia Appendix 1 [file publichealth_v6i3e20828_app1.png]

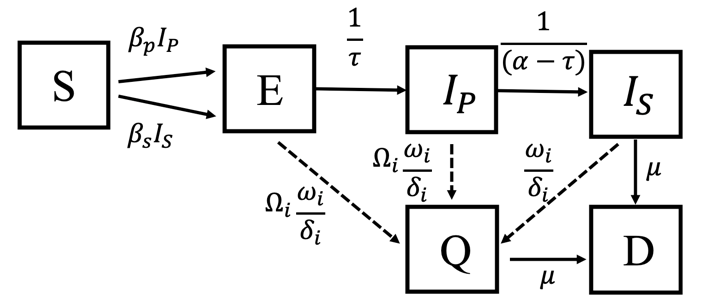

Supplement: Multimedia Appendix 2 [file publichealth_v6i3e20828_app2.png]
